# A Prospective Observational Study Comparing the Efficacy and Safety of Duloxetine and Pregabalin in Diabetic Peripheral Neuropathic Pain

**DOI:** 10.7759/cureus.28683

**Published:** 2022-09-01

**Authors:** Islam Shah, Wiqas Ahmad, Muhammad Islam, Bakhti Jan, Ejaz Ul Haq, Jawad Mahmood, Nasir Iqbal, Mustaqeem Shah

**Affiliations:** 1 Internal Medicine, District Headquarter (DHQ) Hospital, Daggar, PAK; 2 Internal Medicine, Khyber Teaching Hospital, Peshawar, PAK; 3 Gastroenterology and Hepatology/Internal Medicine, Hayatabad Medical Complex, Peshawar, PAK; 4 Medicine, Khyber Medical University, Peshawar, PAK; 5 Adult Cardiology, National Institute of Cardiovascular Diseases, Karachi, PAK; 6 Gastroenterology and Hepatology, Hayatabad Medical Complex, Peshawar, PAK; 7 Gastroenterology and Hepatology, Lady Reading Hospital, Peshawar, PAK

**Keywords:** efficacy, pregabalin, duloxetine, peripheral diabetic neuropathy, diabetes-related peripheral neuropathy

## Abstract

Background and aims

Peripheral neuropathy is a frequent complication of long-standing diabetes mellitus that adversely affects the quality of life. Pregabalin (anticonvulsant) and duloxetine (antidepressant) are often prescribed for diabetic peripheral neuropathic pain. This study aimed to determine and compare the efficacy and safety of pregabalin and duloxetine in patients with diabetic peripheral neuropathic pain.

Materials and methods

This prospective observational study was conducted at District Headquarter (DHQ) Hospital, Daggar, Buner district, Pakistan, from February 15 to July 15, 2022, after approval from the Institutional Research and Ethical Review Board. Confirmation of diabetic peripheral neuropathy was based on the history of diabetes mellitus and vibration perception threshold (VPT) using a biothesiometer. The cut-off was set at 15 volts. VPT of more than 15 volts was considered confirmatory for peripheral neuropathy. Patients were divided equally into two groups. Baseline visual analog scale (VAS) score was recorded for all patients. Tablet pregabalin 300 mg daily was administered for four weeks to one group, while tablet duloxetine in 60 mg strength daily was administered to the other group. VAS score after four-week treatment was recorded and compared. Adverse events experienced by the patient were also noted.

Results

A total of 86 patients were enrolled. The patient ages ranged from 30 to 80 years. Baseline characteristics, including mean age, mean BMI, and mean disease duration of duloxetine versus pregabalin group, were 50.30 ± 8.55 versus 48.20 ± 8.99 years, 23.47 ± 1.23 versus 23.10 ± 1.59 kg/m2 and 21.64 ±7.41 versus 20.04±6.37 months respectively. Duloxetine effectively controlled peripheral neuropathic pain in 81.4% of patients compared to pregabalin in 74.4% of patients. Severe drug-related adverse reactions were observed in 4.6% of patients with duloxetine compared to 0% with pregabalin.

Conclusion

Duloxetine and pregabalin effectively reduce diabetes-related peripheral neuropathic pain. However, duloxetine has slightly better outcomes than pregabalin. The safety profile of pregabalin is better than duloxetine.

## Introduction

Diabetes mellitus, often referred to as the epidemic of the 21st century, is a significant public health problem. As of 2019, the global prevalence of diabetes mellitus worldwide was approximately 9.3%, with a large portion of the population having asymptomatic impaired glucose tolerance [1,2]. The condition is even alarming in Pakistan. The estimated prevalence of diabetes mellitus is 11.7%, with a male-to-female distribution of 1.2:1 [3]

The clinical spectrum of diabetes-related complications is vast [4]. Persistently raised glucose levels potentially lead to anatomical and physiological defects in the body, resulting in diabetes-related complications commonly grouped as microvascular and macrovascular complications [5]. Diabetes-related peripheral neuropathy is one of the microvascular complications, which is a major cause of disability [6]. Diabetic neuropathy is characterized by a burning and tingling sensation in the lower extremities, usually worse at night. The pain affects the sleep pattern and reduces productivity and quality of life [7]. It is suggested that neuropathy is a cumulative effect of microvascular changes in the neuronal feeding artery accentuated by dysmetabolism and possible autoimmune mechanisms [8]. Pregabalin (PGB) and duloxetine (DLX) are frequently prescribed for the symptomatic relief of diabetic neuralgia [9,10]. PGB is an anticonvulsant that acts by inhibiting the pre-synaptic release of excitatory neurotransmitters [11]. DLX is an anti-depressant serotonin and norepinephrine reuptake inhibitor [7]. No head-to-head comparative assessment of the safety and efficacy of PGB and DLX has been done in our local population to manage peripheral diabetic neuropathy.

## Materials and methods

Objectives and setting

This study aims to determine and compare the efficacy and safety of PGB and DLX in diabetic peripheral neuropathic pain. This prospective observational study was conducted at the Department of Internal Medicine, District Headquarter (DHQ) Hospital, Daggar, Buner district, Pakistan, from February 15 to July 15, 2022, after approval from its Institution Research and Ethical Review Board (approval number: 013/2022)

Sampling

Diagnosis of diabetic peripheral neuropathy was confirmed based on history (patients with a history of diabetes for six or more than six months, taking insulin or oral hypoglycemic, complaining of numbness, burning, and tingling sensation with pain in the extremities that worsens at night). Confirmation of peripheral neuropathy was based on vibration perception threshold (VPT) using a biothesiometer. The cut-off for peripheral neuropathy was set at 15 volts. VPT 15 volts or below was considered normal, 16 to 25 volts was called grade 1 (mild) neuropathy, and more than 25 volts was called grade 2 (severe) neuropathy. Both male and female patients aged 30 to 80 years with diabetic peripheral neuropathic pain (visual analog scale (VAS)>4) were included. Patients with type 1 diabetes, diabetic foot ulcer, patients with a history of peripheral vascular diseases, or those who were already taking PGB or DLX were excluded.

Data collection and analysis

A total of 86 patients with diabetic peripheral neuropathy were recruited. Patients were arbitrarily allocated into two groups. Forty-three patients were allocated to the PGB group, and 43 were allocated to the DLX group. PGB and DLX were administered orally in a dose of 300 mg and 60 mg, respectively. Efficacy in terms of symptom relief was plotted on the VAS, with a VAS 0 referring to no pain and 10 to severe pain. Baseline VAS was noted on the day of treatment initiation and then after four weeks of treatment. VAS 0 and 1 were called significant improvements. Safety was assessed in terms of unwanted events experienced by study participants during treatment. These were grouped as mild (nausea, vomiting, decreased appetite, insomnia or somnolence), moderate (visual blurring, ataxia, diplopia peripheral edema, blood pressure changes), and severe (biochemical changes such as elevated liver function tests (LFTs), renal function tests (RFTs) leading to discontinuation of treatment.

Data was recorded using the statistical analysis program IBM SPSS Statistics for Windows, Version 23.0 (Released 2015; IBM Corp., Armonk, New York, United States). Mean ± standard deviation was computed for quantitative variables. For qualitative variables, frequencies and percentages were calculated. Statistical tests of significance student t-test were applied for comparing means of continuous variables while Chi-square test was applied for categorical variables. P-value ≤0.05 was considered statistically significant.

## Results

The study participants were in the age group of 30-80 years, with age of 50.30 ± 8.55 years for the DLX group and 48.20 ± 8.99 years for the PGB group. A mean BMI of 23.47± 1.23kg/m^2^ and 23.10 ± 1.59 kg/m^2^ was calculated in the DLX and PGB groups, respectively. Table 1 depicts the baseline characteristics of patients in both groups.

**Table 1:** Baseline characteristics of patients of both groups DLX: duloxetine; PGB: pregabalin; BMI: body mass index; HbA1c: glycated hemoglobin

Parameters	DLX group			PGB group		
	Minimum	Maximum	Mean ± SD	Minimum	Maximum	Mean ± SD
Age (years)	35	80	50.30±8.55	31	68	48.20±8.99
BMI (kg/m^2^)	21.2	25.3	23.47±1.23	20.0	25.9	23.10±1.59
Disease Duration (months)	10	36	21.64±7.41	11	30	20.04±6.37
HbA1c	7.9	10.3	9.19±0.78	7.1	9.9	8.7±0.81

The mean baseline VAS of the DLX group was 7.3±0.9, while post-treatment VAS was 1.6±0.3. The mean baseline and post-treatment VAS in the PGB group were 6.8±0.5 and 1.9±0.4, respectively, as shown in Table 2.

**Table 2:** Mean VAS score comparison among pregabalin and duloxetine groups VAS: visual analog scale; DLX: duloxetine; PGB: pregabalin

VAS score	DLX group	PGB group
Baseline VAS	7.3±0.9	6.8±0.5
VAS at week 4	1.6±0.3	1.9±0.4

The observed efficacy of DLX after four weeks of treatment was 81.4%, while PGB was effective in reducing diabetic neuropathic pain in 74.4% of patients, as shown in Table 3.

**Table 3:** Comparative efficacy of duloxetine versus pregabalin VAS: visual analog scale; DLX: duloxetine; PGB: pregabalin

VAS 0/1 at week 4	DLX group	PGB group
YES	35 (81.4%)	32 (74.4%)
NO	08 (18.6%)	11 (25.6%)

None of the factors, including age, gender, BMI, disease duration, and HbA1c, significantly affected the efficacy (p-value >0.05).Tables 4, 5 shows subgroup analysis of patients in the PGB and DLX groups, respectively.

**Table 4:** Sub-group analysis of patients in pregabalin group BMI: body mass index; HbA1c: glycated hemoglobin

Parameter		Efficacy		Total	p-value
		YES	NO		
Age	30-45 years	12 (80.0%)	03 (20.0%)	15 (100.0%)	0.214
	46-60 years	16 (80.0%)	04 (20.0%)	20 (100.0%)	
	61-80 years	04 (50.0%)	04 (50.0%)	08 (100.0%)	
Gender	male	19 (76.0%)	06 (24.0%)	25 (100.0%)	0.779
	female	13 (72.2%)	05 (27.8%)	18 (100.0%)	
Disease duration	Less than 20 months	23 (74.2%)	08 (25.8%)	31 (100.0%)	0.956
	More than 20 months	09 (75.0%)	03 (25.0%)	12 (100.0%)	
HbA1c	≤8	13 (76.5%)	04 (23.5%)	17 (100.0%)	0.803
	>8	19 (73.1%)	07 (26.9%)	26 (100.0%)	
BMI	≤23Kg/m^2^	16 (76.2%)	05 (23.8%)	21 (100.0%)	0.861
	>23kg/m^2^	17 (73.9%)	06 (26.1%)	23 (100.0%)	

**Table 5:** Sub-group analysis of patients in duloxetine group BMI: body mass index; HbA1c: glycated hemoglobin

Parameters		Efficacy		Total	p value
		Yes	No		
Age	30-45 years	12 (85.7%)	02 (14.3%)	14 (100.0%)	0.877
	46-60 years	15 (78.9%)	04 (21.1%)	19 (100.0%)	
	61-80 years	08 (80.0%)	02 (20.0%)	10 (100.0%)	
Gender	male	25 (86.2%)	04 (13.8%)	29 (100.0%)	0.243
	female	10 (71.4%)	04 (28.6%)	14 (100.0%)	
Disease duration	Less than 20 months	21 (87.5%)	03 (12.5%)	24 (100.0%)	0.247
	More than 20 months	14 (73.7%)	05 (26.3%)	19 (100.0%)	
HbA1c	≤8%	09 (69.2%)	04 (30.8%)	13 (100.0%)	0.177
	>8%	26 (86.6%)	04 (13.1%)	30 (100.0%)	
BMI	≤23Kg/m^2^	21 (84.0%)	04 (16.0%)	25 (100.0%)	0.604
	>23kg/m^2^	14 (77.7%)	04 (22.3%)	18 (100.0%)	

Regarding the safety profile, PGB was comparatively safer than DLX. Nausea, vomiting, and epigastric discomfort were the most common adverse events reported with DLX while somnolence and dizziness were more common with PGB. Adverse events leading to treatment withdrawal were experienced by two patients (4.6%) in the DLX group. Table 6 shows a comparison of safety profiles between the DLX and PGB groups.

**Table 6:** Comparative safety profile of duloxetine versus pregabalin DLX: duloxetine; PGB: pregabalin

Adverse effects	DLX group	PGB Group
Mild	09 (20.9%)	05 (11.6%)
Moderate	02 (4.6%)	04 (9.3%)
Severe	02 (4.6%)	0 (0.0%)

## Discussion

Peripheral neuropathy is a frequent complication of long-term uncontrolled diabetes mellitus. The pathogenesis of diabetic peripheral neuropathic pain is attributed to the ion channel changes, which affect the function of nerve cells to transmit pain signals. The pain is debilitating and adversely affects the quality of life. Moreover, other complications are also attributed to it, including diabetic foot ulcers and diabetic foot deformities [12,13]. DLX is an antidepressant that reduces neuropathic pain by increasing nor-epinephrine and 5-hydroxytryptamine (5-HT) levels in the CNS. On the other hand, PGB is an anticonvulsant that reduces diabetic peripheral neuropathic pain via inhibition of pain sensors, increasing the pain threshold, and inhibiting the calcium ion channels in the postsynaptic dorsal root pain fibers [14].

This prospective observational study comparing the efficacy of DLX and PGB was the first of its kind in our population. In this study, DLX was observed to reduce diabetes-related peripheral neuropathic pain in 81.4% of patients as compared to 74.4% efficacy of PGB but the results were not significant as the p-value was greater than 0.05. Both DLX and PGB were found to reduce diabetic peripheral neuropathic pain. However, the safety profile of PGB was better than that of DLX. 

In a study by Joharchi and colleagues, DLX and PGB were found equally effective in reducing diabetes-related peripheral neuropathic pain. However, DLX was associated with more severe adverse reactions as compared to PGB [15]. Our study results are in agreement with their observations. In our study, DLX was more effective than PGB in reducing the neuropathic pain in diabetes; however, the difference was not statistically significant. Similar findings regarding the efficacy of DLX and PGB in diabetic peripheral neuropathic pain were reported by Shahid et al. [16]. Goldstein and colleagues compared the dose-related efficacy of DLX for peripheral neuropathic pain. Their findings included a failure of improvement after the administration of 20 mg DLX. However, DLX in 60 mg and 120 mg strength were equally effective, and no clinically significant difference was observed [17].

The observations regarding the safety profile of DLX are variable. A meta-analysis by Quilici and colleagues, comprising 11 studies comparing the safety and efficacy of DLX, PGB, gabapentin, and amitriptyline, observed that DLX was better tolerated as compared to other drugs administered for peripheral neuropathic pain [17]. This is in contrast to the observation of our study. It may be because most of the studies included in the meta-analysis were placebo-based trials and did not directly compare the two drugs for an adverse effect profile. However, that is highlighted in our study.

Our study has limitations like a small sample size and the descriptive nature of the study. Therefore, randomized controlled trials with a large sample size should be performed to better assess the efficacy and safety of DLX and PGB in our population.

## Conclusions

DLX and PGB effectively improve diabetes-related peripheral neuropathic pain; however, DLX has better efficacy (81.4%) compared to PGB (74.4%) in reducing diabetes-related peripheral neuropathic pain. In contrast to efficacy profile, safety profile of PGB is better than DLX. Patients taking DLX for diabetic peripheral neuropathy should be monitored for adverse effects. 
